# Tungsten Molecular Species in Deuterium Plasmas in Contact with Sputtered W Surfaces

**DOI:** 10.3390/molecules29153539

**Published:** 2024-07-27

**Authors:** Gheorghe Dinescu, Cristina Craciun, Silviu Daniel Stoica, Catalin Constantin, Bogdana Maria Mitu, Tomy Acsente

**Affiliations:** 1 National Institute for Lasers, Plasma and Radiation Physics, 409 Atomistilor Str., 077125 Magurele, Romania; dinescug@infim.ro (G.D.); cristina.craciun@inflpr.ro (C.C.); catalin.constantin@inflpr.ro (C.C.); mitu.bogdana@inflpr.ro (B.M.M.); tomy@infim.ro (T.A.); 2 Doctoral School of Physics, Faculty of Physics, University of Bucharest, 405 Atomistilor Str., 077125 Magurele, Romania

**Keywords:** tungsten plasma species, tungsten molecules, tungsten–deuterium plasma, mass spectrometry, magnetron sputtering, fusion technology

## Abstract

We show that in plasmas generated in deuterium in the presence of sputtered W surfaces, various molecular tungsten species are formed, whose chemical composition depends on the presence of gaseous impurities, namely, nitrogen, oxygen, and hydrogen. A magnetron discharge was used for plasma sustaining, and the species were investigated by mass spectrometry and optical emission spectroscopy. The identified tungsten-containing molecules are described by the chemical formula WO_x_N_y_D_z_H_t_, where x = 0–4, y = 0–3, z = 0–3, t = 0–5. Presumptively, even higher mass tungsten molecular species are present in plasma, which were not detected because of the limitation of the spectrometer measurement range to 300 amu. The presence of these molecules will likely impact the W particle balance and dust formation mechanisms in fusion plasmas.

## 1. Introduction

Tungsten is an intensely studied material, with applications in different domains, including the domestic, industrial, high technology, and fusion technology fields. Due to the plasma capabilities for processing materials with extreme properties, like tungsten, plasmas have widely been used in various preparation steps for tungsten-based materials or devices. The extension of these capabilities is further in view, requiring a deeper investigation of plasma surface interactions, with a focus on tungsten surface modification and tungsten species reactions in the plasma volume.

The knowledge of plasmas interacting with a tungsten surface is essential in fusion technology. Presently, tungsten is considered the only material suitable to withstand the extreme operating conditions in a nuclear fusion reactor divertor [1]. In the divertor region where the extraction of the heat and ashes resulting from the fusion reactions is performed, the local temperatures may exceed 1300 °C. The choice of tungsten as a material for plasma-facing components (PFC) in the divertor region [2] is justified by its low sputtering yield, which minimizes material erosion and core plasma contamination. Compared to carbon alternatives, tungsten’s compatibility with deuterium minimizes adverse chemical reactions and structural changes, preserving the material’s integrity and performance, while its low tritium retention is crucial for safety and fuel management [3]. Additionally, tungsten’s high thermal conductivity ensures efficient heat dissipation, maintaining component integrity under severe conditions [4,5]. Despite those advantages, the use of tungsten in fusion reactors still presents challenges: wall erosion cannot be fully prevented, leading to tungsten species release with effects on tungsten dust formation, as well as redeposition [6,7], phenomena which pose safety hazards [8]. We note that dust in fusion devices may appear due to arching and disruptions but also by sputtering and agglomeration [9]. The latest two mechanisms are dependent on the number of impurities present in plasma, and it is possible to be enhanced during the divertor detached regime when gases like Ar or N_2_ are deliberately seeded in plasma in view of the radiative cooling of the divertor [10]. 

The investigation of tungsten species formed in plasmas in contact with tungsten surfaces is therefore a necessary step for the advancement both of the engineering applications of tungsten and for the fusion technology. The present paper addresses this problem by applying dedicated tools for the diagnostics of species and processes appearing in such plasmas by mass spectrometry and optical emission spectroscopy, respectively. Mass spectrometry is crucial due to its ability to give detailed information about the composition, structure, and dynamics of the species in plasmas, for example, used in nanomaterials applications [11,12], polymerization processes [13], reactive processing [14], surface modification [15], and magnetron sputtering [16]. There are also studies reporting on the mass spectrometry investigation of Tokamak plasmas [17,18,19]. Comparatively, optical emission spectroscopy (OES) is much simpler and less costly, but it is limited to the identification of radiative species, and mainly small mass atomic species or diatomic molecules can be observed. Still, being a non-perturbative and easy-to-apply diagnostic, it is widely used for the investigation of plasma parameters and elemental componence in Tokamak studies [20,21,22].

This paper reports, for the first time, the presence of molecular tungsten ionic species with the general chemical formula WO_x_N_y_D_z_H_t_ (x = 0–3; y = 0–2; z = 0–3, t = 0–5) in sputtering plasmas in contact with W surfaces. The experiments were performed in a magnetron sputtering system with a tungsten target. The molecular species were detected and analyzed by mass spectrometry in the mass range 0–300 amu, and the information is complemented by optical emission spectroscopy in the spectral range 200–800 nm. We first indicate the presence of three unknown groups of peaks in the high mass region of the mass spectra recorded in D_2_ plasmas in the presence of Ar sputtering. The assignment of the observed groups to tungsten molecular species was performed using dedicated experiments in deuterium plasmas admixed besides argon, with oxygen, or nitrogen, and with hydrogen being present as an inherent impurity. We conclude with a short discussion on the processes responsible for the species formation and the relevance of results regarding the fusion research.

## 2. Experimental Setups and Methods

### 2.1. Generation of W Plasma and Characterization Techniques

**Figure 1:**
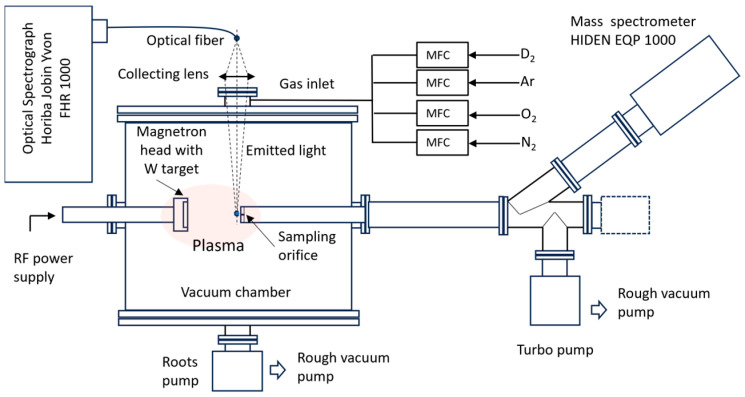
Schematic view of the setup used for plasma generation and plasma investigation by mass spectrometry and optical emission spectroscopy.

The setup used for plasma generation and diagnosis is presented in Figure 1. The magnetron plasma is generated by an RF generator (13.56 MHz), using a typical power of 100 W in a cylindrical vacuum chamber pumped out by a mechanical pump up to a base pressure of 8 × 10^−3^ mbar. The gases (Ar, D_2_, N_2_, O_2_) were injected through dedicated mass flow controllers (MFC) in the magnetron head vicinity, at flow rates in the range of 0–5 sccm. The flow rates were selected to assure inside the chamber a pressure value of 0.07 mbar, compatible with the utilization of the mass spectrometry technique and within the range of the pressure values used in fusion experiments.

An Hiden EQP 1000 instrument was employed for the mass spectrometry measurements. The spectrometer head, provided with an orifice of 100 µm diameter, was placed on the same horizontal axis as the magnetron plasma head, at a 6 cm distance from the target. Mass spectra were collected in positive ions mode. In addition, energy distribution measurements at fixed ion mass were performed for the main species related to deuterium, argon, oxygen, and nitrogen gases, as well as tungsten. Subsequently, we fixed the energy close to the maximum of the energy distribution function of the measured species, at 13 eV (D_2_/Ar), 15 eV (D_2_/Ar/O_2_), and 28 eV (D_2_/Ar/N_2_), thus maximizing the recorded mass spectra signals. 

The OES investigation system consisted of a focusing lens, optical fiber, and an Jobin-Yvon FHR 1000 spectrograph with a focal length of 1000 mm. In the experimental arrangement, the optical spectra are collected vertically from a zone situated in the proximity of the mass spectrometer sampling orifice. Spectra were recorded using a slit size of 20 µm and a grating with 2400 lines/mm.

### 2.2. Mass Spectra Processing Methodology

In our work, we considered the major four W isotopes with high abundances: ^182^W (26.50%), ^183^W (14.31%), ^184^W (30.64%), and ^186^W (28.43%), and the averaged mass of 183.84 amu. As will be exemplified below, the closed values of the isotope mass combined with the limited resolution of the spectrometer causes the superposition of the isotope peaks, leading to specific fingerprints corresponding to tungsten species distributed in the mass range 180–250 amu. 

The method used to fit the group of W^+^ and WH^+^ peaks from the mass spectra recorded on W/Ar/H_2_ plasma was presented in detail in our previous paper [23] and then extended for the case of other WO_x_N_y_H_z_ species in [24]. The asymmetrical shape of the peak profile, the displacement of the peaks from the expected position on the mass scale, and the large width that also varies along the mass scale are the main difficulties in the interpretation of the experimental data, as recorded with the mass spectrometer. In our method, we use the convolution between a Gaussian and an oblique hat function to describe the profile of a mass peak. The mass displacement was corrected through a mass calibration of the spectra, and the width of peaks in any place on the mass scale was determined before the fitting. 

Herewith, by performing the experiments with deuterium and applying the same fitting procedure, we identified the tungsten molecular species appearing in deuterium plasma in contact with W surfaces in the presence of oxygen, nitrogen, and hydrogen gases. These molecules are described by the chemical formula WO_x_N_y_D_z_H_t_, where a single W atom is bonded with various numbers of O, N, D, and H atoms. Their mass peaks are organized in groups, as follows: a group consisting of W isotopes and W isotopes bonded to D and/or H atoms (WD_z_H_t_) with peaks distributed between 180 and 189 amu; a group corresponding to species containing the W atom and one N or O atom and various numbers of D and H atoms (WND_z_H_t_, WOD_z_H_t_) with peaks distributed between 194 and 206 amu; a group corresponding to the W atom bonded to 2 N atoms, or 2 O atoms or one N and one O atom and various numbers of D and H atoms with peaks distributed between 208 and 220 amu; and a group containing 3 N atoms, or 3 O atoms, or N_2_O or O_2_N and various numbers of H or D atoms with peaks distributed between 228 and 240 amu. Accordingly, we divided the high-mass spectra in Ar/D_2_/N_2_ and Ar/D_2_/O_2_ into four regions and fit them separately.

For the processing of each of those groups, we used sets of four peak functions with area proportional to the natural abundance of the four considered W isotopes, with the number of sets being equal with the number of W-containing ionic species supposed to exist in the respective mass range. The simplest example is the mass range 180–190 amu containing W, WH, and WD isotopes, which can be fitted with three sets of four peak functions.

## 3. Results

### 3.1. Mass Spectra of W/D_2_ and W/Ar/D_2_ Plasmas in the Presence of Impurities 

**Figure 2:**
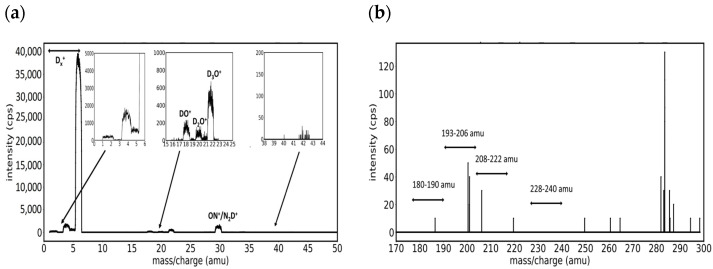
Mass spectra in sole D_2_ (5 sccm): (**a**) spectral range 0–50 amu, and (**b**) spectral range 180–300 amu, where peaks of molecular W species are expected.

The mass spectrum recorded in the positive ions mode on a magnetron plasma generated with a tungsten target in an atmosphere of pure deuterium at 0.07 mbar is presented in Figure 2a for the mass range of 0–50 amu and in Figure 2b for the mass range of 180–300 amu. In the low mass region, the wellseparable peaks correspond to the deuterium-related ions (D^+^, D_2_^+^, D_2_H^+^, D_3_^+^), from which the most present is D_3_^+^ at 6 amu, and to main gaseous impurities (H_2_O^+^ and OD^+^ at 18 amu, D_2_O^+^ at 20 amu, D_3_O^+^ or ND_4_^+^ at 22 amu, and ON^+^ or N_2_D^+^ at 30 amu). The signal corresponding to the W isotopes is expected in the mass interval of 180–190 amu, but the high mass spectrum from Figure 2b presents only some random spikes that are not large enough to be considered peaks. This behavior is caused by the low sputtering yield of the W target by deuterium and the small numbers of impurity ions. 

**Figure 3:**
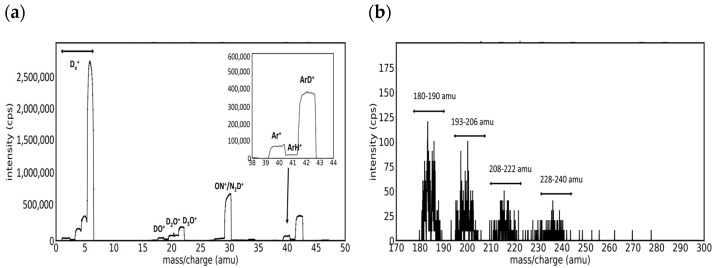
Mass spectra on Ar/D2 (1:5) plasma presenting (**a**) peaks from the low mass region, and (**b**) the groups of peaks with specific form from the high mass region.

The introduction of argon in the magnetron discharge chamber produces an enhancement of the sputtering, as can be seen in Figure 3, where we present the mass spectra recorded on a plasma generated in a mixture of Ar and D_2_ with a ratio of 1:5 (sccm). Like in Figure 2, we present the low mass region (0–50 amu) in Figure 3a and the high mass region (180–300 amu) in Figure 3b. The major difference between the low mass spectra in D_2_ and Ar/D_2_ is the apparition of the peaks of the Ar^+^ and ArD^+^ ions in the mass interval of 40–42 amu, caused by the presence of Ar in the discharge. In the high-mass region, the differences are more noticeable, and one can observe four zones of enhanced signal in the intervals: 180–190 amu, 193–206 amu, 208–222 amu, and 228–240 amu. The similarities between it and the mass spectrum recorded in Ar/H_2_ [24] suggest the formation of molecular tungsten ionic species where hydrogen was replaced by deuterium. Accordingly, we assigned the first zone to W and WD ions, while we presume that the other zones correspond to the W molecular combination with atoms coming from impurities and deuterium. Nevertheless, the high mass spectrum from Figure 3a cannot be fitted with satisfying accuracy, and the ionic species present in plasma cannot be surely identified because the signal was not high enough, indicating that the number of O_2_ and N_2_ impurity molecules species available to react with the sputtered W atoms was still low. 

### 3.2. Mass Spectra of W/D_2_ Plasmas Intentionally Injected with Ar, N_2_, and O_2_ Gases

**Figure 4:**
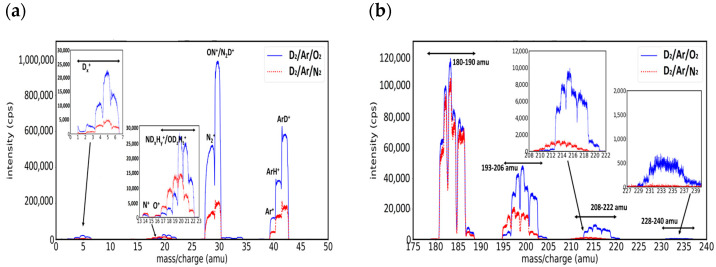
Mass spectra on Ar/D_2_/O_2_(0.5:5:0.5) plasma—blue; and Ar/D_2_/N_2_(0.5:5:0.5) plasma—red; presenting (**a**) peaks from the low mass region and (**b**) the groups of peaks from the high mass region.

In the following, we present the mass spectra recorded from magnetron plasma when D_2_ and Ar gases (5 sccm and 0.5 sccm, respectively) were admixed with 0.5 sccm N_2_ or O_2_ (gas molecules normally appearing as impurities due to air leaks). As an inherent impurity, a small quantity of hydrogen is always present in the magnetron chamber. The Ar role is to deliver W species in plasma by target sputtering, while the deuterium, nitrogen, and oxygen atoms feed the reaction channels leading to the formation of molecular W species. The mass spectra in Ar/D_2_/N_2_ (0.5:5:0.5 sccm) and in Ar/D_2_/O_2_ (0.5:5:0.5 sccm) are presented in Figure 4a,b for the mass intervals 0–50 amu and 175–240 amu, respectively.

The low mass region (Figure 4a) presents features similar to those of Ar/D_2_ spectra (Figure 3a) but enriched with peaks corresponding to species containing N and O atoms. In the high mass region, compared to the Ar/D_2_ plasma, the mass spectra recorded in the presence of N_2_ and O_2_ show high intensity and well-delimited groups of peaks, with similar shapes. The fingerprint of the four groups of peaks in the high mass region of the Ar/D_2_/N_2_ and Ar/D_2_/O_2_ spectra was like that observed in the case of Ar/H_2_/N_2_ and Ar/H_2_/O_2_ plasma [24], and we used the same approach for spectra fitting and species identification, discussing separately each of the groups.

**Group A. Mass peaks in the range of 180–190 amu**

**Figure 5:**
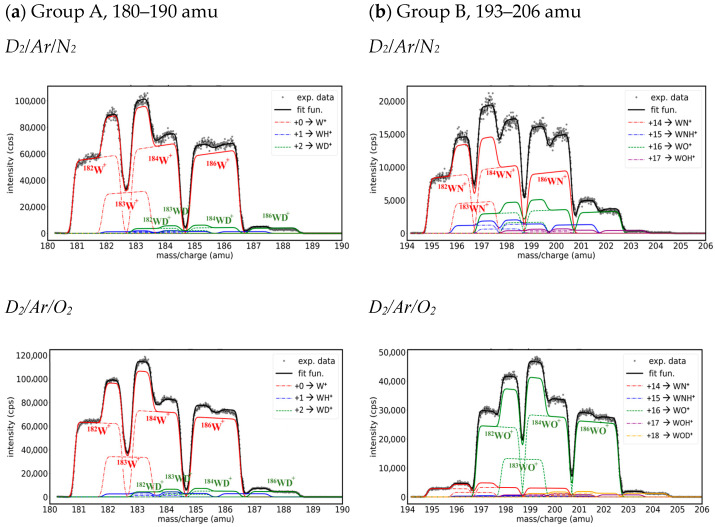
Groups of fitted peaks from the mass spectra on Ar/D_2_/N_2_ (top) and Ar/D_2_/O_2_ (bottom) (0.5:5:0.5) plasma: (**a**) 180–189 amu, (**b**) 193–206 amu. The legend contains the displacement with respect to W isotopes and the W species that are most probable for each gas mixture.

In the 180–190 amu mass interval, the shape of the group of peaks (Figure 5a) was almost identical in Ar/D_2_/N_2_ and Ar/D_2_/O_2_ and like that observed in Ar/H_2_ [24]. The most intense signal came from the four superposed peaks corresponding to the W isotopes, and the small intensity tail from the higher mass can be associated with the presence of WD_+_, while the WH^+^ ions had a small, almost negligible, contribution.

**Group B. Mass peaks in the range of 193–206 amu**

The second group of peaks was observed in the region of 194–206 amu (Figure 5b). The differences between Ar/D_2_/N_2_ and Ar/D_2_/O_2_ spectra were noticeable: the main signal in Ar/D_2_/N_2_ came from WN^+^, while in Ar/D_2_/O_2_, it came from WO^+^. In both cases, the presence of hydride ions was observed (WNH^+^ at +15 amu and WOH^+^ at +17 amu), and the formation of deuterium species was evidenced in the case of Ar/D_2_/N_2_ by the high intensity of the +16 amu peak, which included contributions from two species, WND^+^ and WO^+^; similarly, in the case of Ar/D_2_/O_2_, this was by the existence of peaks at +18 amu that corresponded to WOD^+^. We neglected the possibility of WNH_2_^+^ and WOH_2_^+^ formation as we did not observe their peaks in Ar/H_2_ discharge [24].

**Group C. Mass peaks in the range of 208–222 amu**

**Figure 6:**
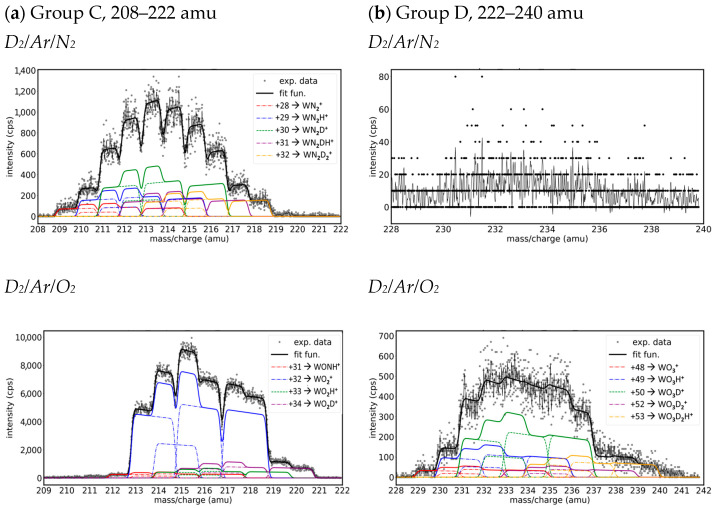
Groups of fitted peaks from the mass spectra on Ar/D_2_/N_2_ (top) and Ar/D_2_/O_2_ (bottom) (0.5:5:0.5) plasma: (**a**) 208–222 amu, and (**b**) 228–240 amu. The legend indicates the displacement with respect to W isotopes and the W species that are most probable for each gas mixture.

The identification of the molecular species from the third group (208–222 amu, Figure 6a) is difficult as there are more possible species with the same mass. As in the case of Ar/H_2_ plasma in the presence of O_2_ and N_2_ impurities from our previous work [24], the third group of Ar/D_2_/N_2_ presented more sets of peaks with similar intensities, while in the case of Ar/D_2_/O_2_, the signal came mainly from one high-intensity set of peaks that probably have contributions from more different species. The sets of peaks that appear at odd mass differences (+29, +31, +33 amu) must contain hydrogen as all the rest of the atoms had even mass. Because the difference between the mass of the oxygen and nitrogen atoms is the same as the mass of deuterium, for each set of peaks, with an even mass difference, we expected contributions from more species of the form WN_2_D_x_, WNOD_x−1_, and WO_2_D_x−2_. For example, in the case of Ar/D_2_/N_2_, the highest set of peaks was placed at +30 amu and was able to have a contribution from both WN_2_D and WNO, while in the case of Ar/D_2_/O_2_, the highest set from +32 could contain three species: WN_2_D_2_, WNOD, and WO_2_.

**Group D. Mass peaks in the range of 228–240 amu**

The last group can be found in the range of 228–240 amu (Figure 6b) for the case of Ar/D_2_/O_2_ spectra. As in the case of hydrogen plasma [24], in deuterium plasma impurified with a small amount of argon and nitrogen, the fourth group of peaks had too low intensity to be fitted with satisfying accuracy. The first set of peaks that appeared in the case of Ar/D_2_/O_2_ spectra was placed at a mass difference of +48 amu and it corresponded to the WO_3_^+^ ions. Because we did not observe species that contain nitrogen in Ar/D_2_/N_2_, we did not expect this kind of species in Ar/D_2_/O_2_ and considered only W molecules that contain three atoms of oxygen and one or more atoms of hydrogen and deuterium.

A synoptic view of the identified species, distributed with their mass across all presented peak groups, is presented in Table 1. We underline that the presence of ionic species WO_x_N_y_D_z_H_t_^+^, as revealed by mass spectrometry, also indicates the presence of their neutral counterparts with the same general form WO_x_N_y_D_z_H_t_. They are the parent molecules of the ionic species. As is discussed next section, the ionic species are predominately formed in electronic collisions with plasma electrons. 

## 4. Discussion of the Mechanisms of Formation of Tungsten Molecular Species

The complete chemistry of W/Ar/D_2_ magnetron sputtering plasma in low-pressure radiofrequency discharge with nitrogen, oxygen, and hydrogen impurities may include a multitude of reactions, but in the following, we present only some processes, which we presume may lead to the formation of the species discussed in this study. The type of process is strongly dependent on the position in the discharge. We start the discussion with surface processes, and we continue with the volume processes. 

As a simplified general view, the plasma contains populations of electrons, as well as heavy particles: ions and neutrals. In the magnetron discharge, the particles evolve in combined magnetic and electric fields. Due to the magnetic field, the most intense plasma is retained in the proximity of the target, a region characterized by high electron and ion densities. While the electrons are repelled by the electric field and trapped in front of the target surface by the magnetic field, the ions are accelerated toward the surface, hitting it, and causing sputtering. The W atoms and W ions are removed from the surface and released in the plasma volume due to bombardment with ions the representative reaction being:

W(surface)+ Ar^+^ (plasma)→ W, W^+^ (plasma)

In addition, sputtering can be produced also due to the bombardment with energetic Ar atoms, if their energy is high enough, over 100 eV [25]. It is worth mentioning that molecular W species can be released directly from the target in particular situations. In the presence of deuterium, as well as reactive gases such as oxygen and nitrogen, the chemistry of the target changes, and on its surface can be formed tungsten hydrides, oxides, nitrides, or other tungsten-containing chemical groups. Such chemical groups can be released into plasma directly from the surface, as proposed for the WD species detected in Tokamaks [26,27]. These surface processes can be favored by the change in the sub-surface material structure. For example, the existence of a D- or H-supersaturated surface layer is accompanied by lattice distortion and microstructural defects in deuterium or hydrogen-plasma-irradiated tungsten [28,29,30]. Direct metal hydride sputtering was also observed from Be/H_2_ plasmas [31]. Similarly, nitrogen can be stored in a thin layer on the tungsten surface during exposure to nitrogen plasma, and a significant part of tungsten is released in plasma as WN molecules [32], however with a decreased sputtering rate compared to a pure tungsten surface [33]. In addition, sublimation of WO_3_ oxide, which starts at 700–800 degrees Celsius [34,35], may occur under ion bombardment at lower temperatures, possibly also being a source of gaseous molecular tungsten species. Nevertheless, even if they cannot be excluded, these processes are much less documented, and it is difficult to be counted at present. 

**Figure 7:**
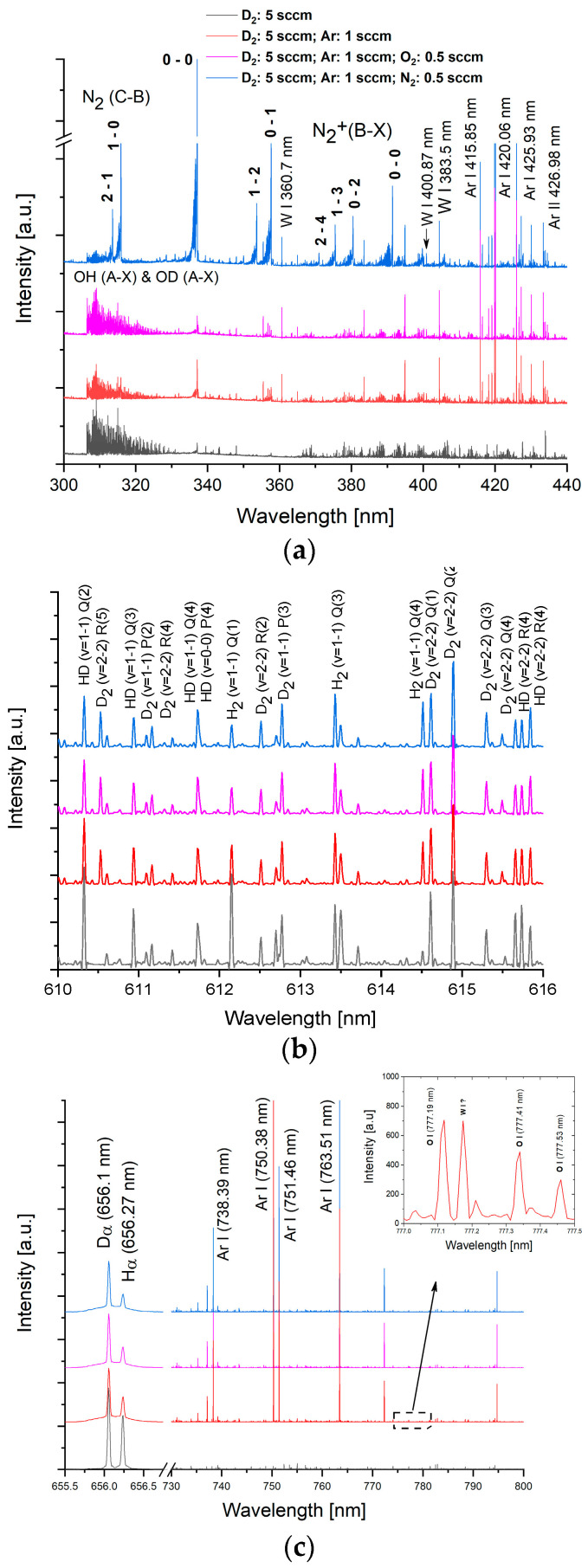
High-resolution optical emission spectra and the assignation of lines and bands to the atomic and molecular species: (**a**) signature of W atoms, OH radicals, and N_2_ molecules; (**b**) Fulcher bands corresponding to D_2_, H_2_, and HD molecules; (**c**) Hα and Dα lines corresponding to the Balmer series of H and D atoms, and the signature of O atoms.

Regarding the volume processes, one must account for the fact that in magnetron plasma, the kinetic energy of electrons, which is described by the temperature of the electronic population, is much higher than that of heavy particles (T_e_ ~ 2–4 eV, compared to T_i_, T_n_ ~ 0.03–0.06 eV). The remarkably different values of the temperatures of electrons and heavy particles brought to such plasmas the terminology of non-thermal or non-equilibrium plasmas. The kinetic energies of the electrons are comparable with the energy thresholds for dissociation, excitation, and ionization. Accordingly, atomic radicals, as well as excited and ionized species, are formed by via inelastic energy transfer by electronic collisions with heavy particles. Generically, if we denote by A an atom and AB a molecule, the electronic collision processes can be described by e + A → e + A* (atomic excitation); e + AB→ e + AB* (molecular excitation); e + A → e + A^+^ + e (atomic ionization); e + AB → e + AB ^+^ + e (molecular ionization); e + AB → e + A + B (dissociation). Therefore, we will find in plasma, besides the neutral species resulting from target sputtering (W atoms), from gas injection (Ar, D_2_, N_2_, O_2_ species), and from vacuum leaks (O_2_, N_2_, H_2_, H_2_O molecular traces), a large variety of atomic and molecular species resulting from electronic excitation, dissociation, and ionization processes. Examples of such species are as follows: (i) ground states atomic radicals like D, Ar, N, O, H, etc.; (ii) excited atomic and molecular neutrals like Ar*, Ar^m^, D_2_*, N2*, O_2_*, D*, N*, N^m^, O*; H*, etc.; (iii) atomic and molecular ions in ground or excited states, like W^+^, Ar^+^, D^+^, N^+^, O^+^, H^+^, D_2_^+^, N_2_^+^, O_2_^+^, H_2_^+^, etc. Some of these species, namely, the excited ones emitting in the 200–800 nm range, are easily identified in the emission spectra. Representative OES spectra, recorded in various conditions, are presented in Figure 7, where the most important identified species are indicated. More details on the electronic collision processes can be found in [36].

Despite their significantly lower kinetic energy (low values of T_i_, T_n_), excited and ionized heavy particles contain an important internal energy corresponding to their excited and ionized states. Their internal energy is redistributed by heavy particle collisions, leading to chemical reactions, species additions, fragmentation, charge transfer, etc. Part of the ionic species created in this way (Ar^2+^, ArD^+^, D_3_^+^, ND^+^; OD^+^; OH^+^; ArH^+^, D_2_O^+^, HD_2_^+^, etc.) are observed in the low mass region of the mass spectra (Figure 4a). Details on the heavy particle processes involving Ar^+^ ions are found in [37], while in [38] are the presented details of reactions between hydrogen and deuterium species. The reference [39] presents reactions of deuterium and hydrogen molecules with the N^+^ ion. 

Concerning the W element, the reaction rates for ionization by electron impact and collisions with Ar^m^ metastables and Ar^+^ ions leading to the formation of W^+^ or W^2+^ ions are mentioned in [40]. However, the literature is lacking in providing plasma phase reactions of W or W^+^, leading to higher mass molecules, such as those mentioned in Table 1. Herewith, we propose, by considering species revealed by the present experiments, that higher mass species of the type WO_x_N_y_D_z_H_t_^+^ can be formed by associative reactions of lower mass W-containing neutral molecules (reaction A, below) and lower mass W-containing ionic species (reaction B), with molecular species which does not contain W, as follows:

WO_x−a_N_y−b_D_z−c_ + O_a_N_b_D_c_^+^ → WO_x_N_y_D_z_^+^ (reaction A)

with x = 0–3, y = 0–2, z = 0–5, x + y = 3 and a = 0–x, b = 0–y, c = 0–z.

WO_x−a_N_y−b_D_z−c_^+^ + O_a_N_b_D_c_ → WO_x_N_y_D_z_^+^ (reaction B)

with x = 0–3, y = 0–2, z = 0–5, x + y = 3 and a = 0–x, b = 0–y, c = 0–z.

Using these generic reactions, the species from our first group (180–190 amu) were obtained when x + y = 1, those of the second group (193–206 amu) were obtained from x + y = 2, those of the third group were obtained for x + y = 3 for (208–222 amu), and those for the fourth group (228–240 amu) were obtained with x = 4, y = 0. Of course, a dynamic equilibrium was established between the neutral and ionic molecular W-containing species of the same mass sustained by electron ionization and electron-ion recombination processes. 

Finally, the present results do not include species of higher mass, containing, for example, molecules with two or more than two tungsten atoms. This is because the mass spectrometer does not detect species with a mass higher than 300 amu. Still, such species (molecules containing one W atom with an increased number O and N atoms and molecules with more than two W atoms) should exist. First, in several works, the tendency of W surfaces to release W molecular species or clusters with different sizes when exposed to high energy conditions is highlighted. Reactions leading to the formation of high mass tungsten oxide molecules at the surface and in the gas phase in vacuum are discussed in [41]. In early low-pressure oxidation experiments [42], WO_2_, WO_3_, W_2_O_6_, and W_3_O_9_ were detected by mass spectrometry, at temperatures higher than 1400 °C This is explained by the fact that tungsten oxides formed at the surface are volatile, and the oxide molecules formed at the surface evaporate [43]. Moreover, the tendency of W materials to form clusters in plasma volume is substantiated by experiments of synthesis of tungsten oxide nanoparticles in a low-pressure hydrogen/oxygen combustion flame fed with tungsten hexafluoride, where the formation of (WO_3_)_n_ cluster with *n* up to 38 was observed [44]. Herewith, we propose also that tungsten molecules with more than two W atoms can be formed in volume by associative reactions of type A or B, where both reaction partners contain W atoms, a process demonstrated in the case of molecular C species formed in an argon discharge fed with C_2_H_2_, where C_x_H_y_^+^ molecules with carbon numbers up to x = 9 were detected by mass spectrometry [45]. The building up of higher mass W-containing molecules is of the highest interest for fusion technology where tungsten dust formation is an issue [7,46].

## 5. Conclusions

The atomic and molecular species present in plasmas in contact with tungsten surfaces are intensively studied because of their relevance for thermonuclear fusion, as well as the wide range of technological applications of tungsten. Herewith, we performed mass spectrometry and optical emission spectroscopy investigations of deuterium plasmas bordered by a tungsten surface sputtered by argon, in the presence of oxygen and nitrogen gases. The experiments were devoted to the identification of molecules containing tungsten bonded to the respective gas atoms. We show, for the first time, that besides single W ions and the molecular species recently observed by us in impurified W/H_2_ plasmas [24], deuterated tungsten ionic species like WD^+^, WDO^+^, WND^+^, WND_2_, WNOD_2_^+^, WNOD^+^, WO_2_D^+^, WNOD^+^, WO_3_D^+^, and WO_3_D_2_^+^ are present. We propose that such species may come from the target surface due to sputtering or ion bombardment enhanced sublimation or are formed by reactions in the plasma volume. We observed that the species formation processes are enhanced in the presence of Ar or N_2._ In the case of fusion experiments, such gases are intentionally injected to reach the divertor detachment regime.

This research opens the way to extend the research methodologies and to include the molecular tungsten species in the plasma tableau. Up until the present, the amount of tungsten in fusion plasmas was evaluated mainly by optical emission spectroscopy of W atoms and measurements of the material erosion and redeposition. The accounting of the identified molecular W species in the W particle balance in Tokamak will lead to a more detailed and closer to reality description of the fusion plasmas and of plasma–wall interaction phenomena. Moreover, because the identified tungsten molecules (Table 1) and their higher mass equivalents can function as germs for W cluster growth and particle formation, these novel results shed a new light on the tungsten dust formation in Tokamak.

## Figures and Tables

**Table 1:** All the identified W species together with their mass and their mass shifts with regard to the position of the W isotopes.

Regions 180–189 amu and 193–206 amu			Region 208–222 amu			Region 228–240 amu		
Mass Shift (amu)	Centre of the Peak (amu)	Pos. Ions	Mass Shift (amu)	Centre of the Peak (amu)	Pos. Ions	Mass Shift (amu)	Centre of the Peak (amu)	Pos. Ions
0	182,183,184,186	W^+^	28	210,211,212,214	WN_2_^+^	48	230,231,232,234	WO_3_^+^
1	183,184,185,187	WH^+^	29	211,212,213,215	WN_2_H^+^	49	231,232,233,235	WO_3_H^+^
2	184,185,186,188	WD^+^	30	212,213,214,216	WN_2_H_2_^+^ WN_2_D^+^ WNO^+^	50	232,233,234,236	WO_3_H_2_^+^ WO_3_D^+^
14	196,197,188,200	WN^+^	31	213,214,215,217	WN_2_H_3_^+^ WN_2_DH^+^ WNOH^+^	52	234,235,236,238	WO_3_H_4_^+^ WO_3_D_2_^+^
15	197,198,189,201	WNH^+^	32	214,215,216,218	WN_2_H_4_^+^ WN_2_D_2_^+^ WNOH_2_^+^ WNOD^+^ WO_2_^+^	53	235,236,237,239	WO_3_H_5_^+^ WO_3_D_2_H^+^
16	198,199,200,202	WNH_2_^+^ WND^+^ WO^+^	33	215,216,217,219	WN_2_H_5_^+^ WN_2_D_2_H^+^ WNOH_3_^+^ WNODH^+^ WO_2_H^+^			
17	199,200,201,203	WNH_3_^+^ WNDH^+^ WOH^+^	34	216,217,218,220	WN_2_H_6_^+^ WN_2_D_3_^+^ WNOH_4_^+^ WNOD_2_^+^ WO_2_H_2_^+^ WO_2_D^+^			
18	200,201,202,204	WNH_4_^+^ WND_2_^+^ WOH_2_^+^ WOD^+^						

## Data Availability

The original contributions presented in the study are included in the article, further inquiries can be directed to the corresponding authors.
